# 2838. Clinical Application and Validation of a Predictive Antimicrobial Resistance Risk Categorization Framework for Patients with Uncomplicated Urinary Tract Infection

**DOI:** 10.1093/ofid/ofad500.2448

**Published:** 2023-11-27

**Authors:** Ryan K Shields, Wendy Y Cheng, Kalé Kponee-Shovein, Fernando Kuwer, Chi Gao, Ashish V Joshi, Fanny S Mitrani-Gold, Patrick Schwab, Diogo Ferrinho, Malena Mahendran, Daniel Indacochea, Lisa Pinheiro, Jimmy Royer, Madison T Preib, Jennifer Han, Richard Colgan

**Affiliations:** University of Pittsburgh, Pittsburgh, PA; Analysis Group, Inc, Boston, Massachusetts; Analysis Group, Inc., Boston, Massachusetts; Analysis Group, Inc., Boston, Massachusetts; Analysis Group, Inc., Boston, Massachusetts; GlaxoSmithKline plc., Collegeville, Pennsylvania; GlaxoSmithKline plc., Collegeville, Pennsylvania; GSK, Collegeville, Pennsylvania; GSK, Collegeville, Pennsylvania; Analysis Group, Inc., Boston, Massachusetts; Analysis Group, Inc., Boston, Massachusetts; GSK, Collegeville, Pennsylvania; Analysis Group, Inc., Boston, Massachusetts; GSK, Collegeville, Pennsylvania; GlaxoSmithKline, Collegeville, Pennsylvania; University of Maryland School of Medicine, Baltimore, Maryland

## Abstract

**Background:**

Empiric antibiotic (ABX) treatment for uncomplicated urinary tract infections (uUTIs) can be ineffective due to antimicrobial resistance (AMR). Understanding the risk of AMR using data-driven approaches can inform appropriate ABX selection. We developed an AMR pathogen risk categorization framework in *E. coli* caused uUTI using predictive modeling and evaluated its clinical validity.

**Methods:**

Eligible females with uUTI confirmed by positive *E. coli* urine culture treated with nitrofurantoin (NTF), trimethoprim/sulfamethoxazole (SXT), fluoroquinolones (FQs), or beta-lactams (BLs) were identified from the Optum de-identified electronic health record data set (Oct 2015–Feb 2020). We developed predictive models using machine learning to quantify AMR probability for each ABX class. A framework with 3 risk categories (low, moderate, high) was constructed using the predicted probability (PP) of non-susceptibility (NS) (**Table 1**). Six patient profiles from differing risk categorizations were reviewed for clinical validity by 5 clinicians (4 medical doctors, 1 pharmacist).

**Results:**

Of 87,487 eligible patients, approximately half were classified as low or high risk (44.0–49.1% across ABX classes). The proportion of patients with infections due to NS organisms was 5–12-fold higher among patients classified as high or moderate vs low risk (**Figure 1**). After review of the patient profiles (**Table 2**), clinical experts confirmed the consistency of modeled risk classification for all 6 patients with their own assessment of AMR risk across all drug classes. Patient 1 was aged 20 yrs, White, West residence, no UTI or ABX history 1 year prior to her uUTI; PP of NS was low (NTF 1.5%, SXT 16.6%, FQs 4.8%, BLs 8.4%) and was classified as low risk for all ABX classes. In contrast, patient 6 was post-menopausal, Black, Midwest residence, and had UTI episodes, prior AMR, and multiple healthcare visits 1 year prior to uUTI. She had high PP of NS (NTF 10.3%, SXT 97.2%, FQs 96.4%, BLs 48.0%) and was categorized as high risk for all ABX classes.

**Figure:**
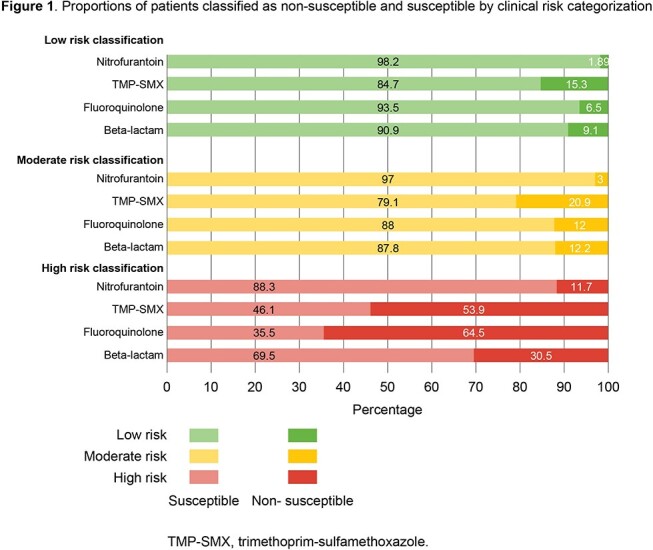

**Figure:**
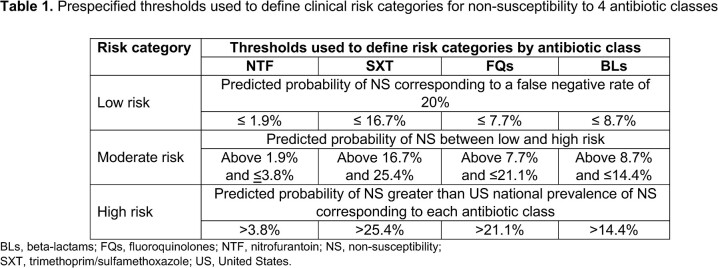

**Figure:**
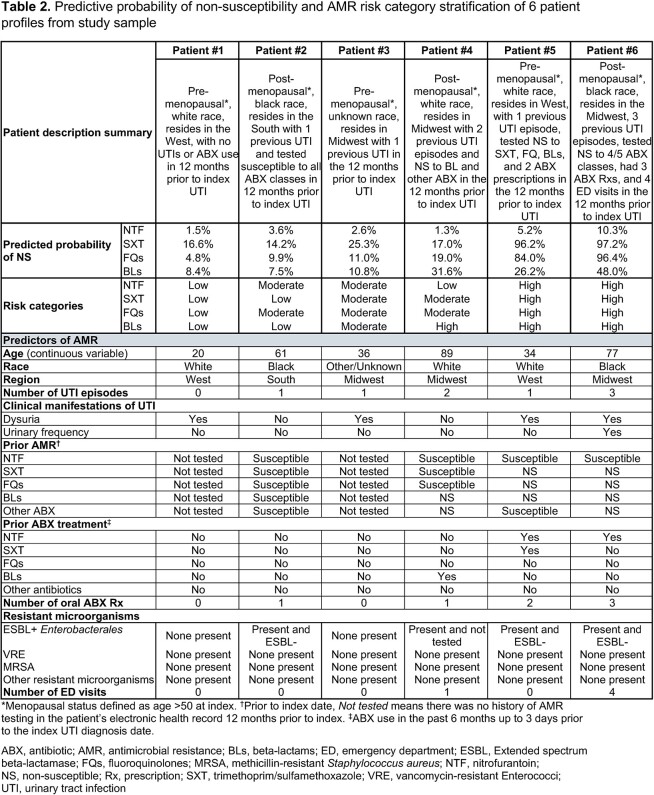

**Conclusion:**

AMR risk varied greatly between patients. Our prediction model contextualizes patients’ AMR PP to 4 commonly prescribed ABX classes in the setting of uUTIs. Clinical application of this framework could inform appropriate empiric ABX selection for patients with uUTI.

**Disclosures:**

**Ryan K. Shields, PharmD, MS**, Allergan: Advisor/Consultant|Cidara: Advisor/Consultant|Entasis: Advisor/Consultant|GSK: Advisor/Consultant|Melinta: Advisor/Consultant|Melinta: Grant/Research Support|Menarini: Advisor/Consultant|Merck: Advisor/Consultant|Merck: Grant/Research Support|Pfizer: Advisor/Consultant|Roche: Grant/Research Support|Shionogi: Advisor/Consultant|Shionogi: Grant/Research Support|Utility: Advisor/Consultant|Venatorx: Advisor/Consultant|Venatorx: Grant/Research Support **Wendy Y. Cheng, MPH, PhD, ORCID: 0000-0002-8281-2496**, Analysis Group, Inc.: Wendy Y. Cheng is an employee of Analysis Group, Inc., a consulting company that received funding from GSK to conduct this study **Kalé Kponee-Shovein, ScD**, Analysis Group, Inc: Employee of Analysis Group, Inc., which received funding from GSK to conduct the study **Fernando Kuwer, MSc**, Analysis Group, Inc.: Employee of Analysis Group, Inc., which received funding from GSK to conduct the study **Chi Gao, ScD**, Analysis Group, Inc.: Employee of Analysis Group, Inc., which received funding from GSK to conduct the study **Ashish V. Joshi, PhD**, GSK: Employee|GSK: Stocks/Bonds **Fanny S. Mitrani-Gold, MPH**, GSK: Employee|GSK: Stocks/Bonds **Patrick Schwab, PhD**, GSK: Employment|GSK: Stocks/Bonds **Diogo Ferrinho, PharmD**, GSK: Employee|GSK: Stocks/Bonds **Malena Mahendran, MS**, Analysis Group, Inc.: Malena Mahendran is an employee of Analysis Group, Inc., a consulting company that received funding from GSK to conduct this study **Daniel Indacochea, PhD**, Analysis Group, Inc.: Employee of Analysis Group, Inc., which received funding from GSK to conduct the study **Lisa Pinheiro, MFin**, Analysis Group, Inc.: Employee of Analysis Group, Inc., which received funding from GSK to conduct the study **Jimmy Royer, PhD**, Analysis Group, Inc.: Employee of Analysis Group, Inc., which received funding from GSK to conduct the study **Madison T. Preib, MPH**, GSK: Employee|GSK: Stocks/Bonds **Jennifer Han, MD**, GSK: Employment|GSK: Stocks/Bonds **Richard Colgan, MD**, GSK: Advisor/Consultant

